# MIR21-induced loss of junctional adhesion molecule A promotes activation of oncogenic pathways, progression and metastasis in colorectal cancer

**DOI:** 10.1038/s41418-021-00820-0

**Published:** 2021-07-05

**Authors:** Andrea Lampis, Jens C. Hahne, Pierluigi Gasparini, Luciano Cascione, Somaieh Hedayat, Georgios Vlachogiannis, Claudio Murgia, Elisa Fontana, Joanne Edwards, Paul G. Horgan, Luigi Terracciano, Owen J. Sansom, Carlos D. Martins, Gabriela Kramer-Marek, Carlo M. Croce, Chiara Braconi, Matteo Fassan, Nicola Valeri

**Affiliations:** 1 Division of Molecular Pathology, Centre for Evolution and Cancer, The Institute of Cancer Research, London, UK; 2 Department of Cancer Biology and Genetics, Comprehensive Cancer Center, The Ohio State University College of Medicine, Columbus, OH USA; 3 School of Biomedical Sciences and Pharmacy, College of Health, Medicine and Wellbeing, University of Newcastle, Newcastle, NSW Australia; 4 Hunter Medical Research Institute, New Lambton Heights, NSW Australia; 5 Bioinformatics Core Unit, Institute of Oncology Research (IOR), Faculty of Biomedical Sciences, Università della Svizzera italiana, Bellinzona, Switzerland; 6 Swiss Institute of Bioinformatics, Bellinzona, Switzerland; 7 Cancer Research UK Beatson Institute, Glasgow, UK; 8 Institute of Cancer Sciences, University of Glasgow, Glasgow, UK; 9 Department of Biomedical Sciences, Humanitas University, Milan, Italy; 10 IRCCS Humanitas Research Hospital, Milan, Italy; 11 Division of Radiotherapy and Imaging, The Institute of Cancer Research, London, UK; 12 Department of Medicine, Surgical Pathology Unit, University of Padua, Padua, Italy; 13 Istituto Oncologico Veneto, Istituto di Ricovero e Cura a Carattere Scientifico, Padua, Italy; 14 Department of Medicine, The Royal Marsden Hospital, London, UK; 15 Division of Surgery and Cancer, Imperial College London, London, UK

**Keywords:** Cancer genomics, Tumour-suppressor proteins, Prognostic markers

## Abstract

Junctional adhesion molecules (JAMs) play a critical role in cell permeability, polarity and migration. JAM-A, a key protein of the JAM family, is altered in a number of conditions including cancer; however, consequences of JAM-A dysregulation on carcinogenesis appear to be tissue dependent and organ dependent with significant implications for the use of JAM-A as a biomarker or therapeutic target. Here, we test the expression and prognostic role of JAM-A downregulation in primary and metastatic colorectal cancer (CRC) (*n* = 947). We show that JAM-A downregulation is observed in ~60% of CRC and correlates with poor outcome in four cohorts of stages II and III CRC (*n* = 1098). Using JAM-A knockdown, re-expression and rescue experiments in cell line monolayers, 3D spheroids, patient-derived organoids and xenotransplants, we demonstrate that JAM-A silencing promotes proliferation and migration in 2D and 3D cell models and increases tumour volume and metastases in vivo. Using gene-expression and proteomic analyses, we show that JAM-A downregulation results in the activation of ERK, AKT and ROCK pathways and leads to decreased bone morphogenetic protein 7 expression. We identify MIR21 upregulation as the cause of JAM-A downregulation and show that JAM-A rescue mitigates the effects of MIR21 overexpression on cancer phenotype. Our results identify a novel molecular loop involving MIR21 dysregulation, JAM-A silencing and activation of multiple oncogenic pathways in promoting invasiveness and metastasis in CRC.

## Introduction

Junctional adhesion molecule A (JAM-A) is a critical component for the maintenance of epithelial cell homoeostasis and preservation of barrier function in the large intestine. In physiological conditions, JAM-A regulates paracellular permeability and controls ionic and water flux protecting against pathogens. Aberrant JAM-A expression has been linked to enhanced colon epithelial permeability, increased inflammation due to leucocyte transmigration and loss of barrier function in pre-clinical models and patients with inflammatory bowel disease [1].

Analyses of JAM-A expression and function in different cancer types including breast [2–4], lung [5] and nasopharyngeal cancers [6], as well as in haematological malignancies [7], have produced conflicting results, suggesting that JAM-A expression and its role in carcinogenesis might be either tissue/organ specific or stage dependent.

In gastrointestinal tumours, loss of JAM-A has been reported as a negative prognosticator in patients with pancreatic [8] and gastric cancers [9]. Pre-clinical data suggest that *Apc* loss induces JAM-A downregulation in the intestine of genetically engineered mouse models (GEMMs) eliciting loss of barrier function, loss of polarity and inflammation [10]. No data are currently available on JAM-A expression in early and metastatic colorectal cancer (CRC). Here, we tested prognostic and functional implications of JAM-A dysregulation, in early and metastatic CRC.

## Materials and methods

### Patients’ TMAs cohorts

The Glasgow Royal Infirmary TMA Cohort (Glasgow, UK) included 266 evaluable core cases composed of 247 stages I–III sporadic CRC and 19 healthy control tissues. The Basel University Hospital Cohort TMA (Basel, Switzerland) included 659 evaluable core cases composed of 644 stages II–III sporadic CRC and 15 healthy control tissues. All patients gave informed consent. Commercial TMA Cohort (CO702a and HLin-Ade075Met-01; US Biomax Inc, Rockville, USA) included a total of 127 evaluable core cases of which 56 were evaluable primary CRC (stages I–IV, CO702a and grades 1–3, HLin-Ade075Met-01), 15 were healthy control tissues and 56 were evaluable CRC metastasis tissues at different sites. Membranous and cytoplasmic JAM-A were measured using the histo-score (*H*-score) to assess the intensity of immunoreactivity as per the following formula: (3 × percentage of strong staining) + (2 × percentage of moderate staining) + (1 × percentage of weak staining), resulting in a range between 0 and 300.

### Cell lines and patient-derived organoids (PDOs)

CRC cell lines were purchased from ATCC. DLD-1 MIR21^wt^ and MIR21^KO^ were a kind gift from Jian Yu (University of Pittsburgh Cancer Institute, Pittsburgh, USA) to CMC (Ohio State University Columbus, USA) and RKO MIR21^wt^ and MIR21^KO^ were purchased from Horizon Discovery (Cambridge, UK) and were cultured in McCoy’s 5A or DMEM modified medium, respectively, (Gibco, Carlsbad, CA, USA) under standard conditions at 37 °C with 5% of CO_2_ in a controlled incubator. All cell lines were supplemented with 10% FBS (Gibco) plus 1% penicillin/streptomycin mixture (Gibco). Human organoids derived from tumour biopsies of metastatic CRC patients were previously described [11]. All cell models were regularly tested for mycoplasma contamination.

### Plasmids and lentiviral infections

TRIPZ lentiviral plasmids for JAM-A silencing (TRIPZ JAM-A sh) and relative control (TRIPZ JAM-A CTRL) were purchased from Thermo Fisher Scientific (Waltham, MA, USA). Lentiviral particles production and target cells transduction was performed as per the manufacturer’s instructions. MIR21-overexpressing vector generation and cell transduction was previously described [12]. Following viral transductions infected cells were selected with puromycin (10 μg/ml Caco-2 and 2 μg/ml DLD-1, 1 μg/ml PDOs) for 2 weeks.

### Minicircle (MC) plasmids

MC plasmids (System Biosciences, Palo Alto, CA, USA) were generated as per the manufacturer’s instructions. Fragments obtained by in-fusion cloning were used in order to generate MC-JAM-A CDS WT 3′-UTR and MC-JAM-A CDS Δ21 3′-UTR. Amplicons containing fusion products were PCR amplified by means of Phusion high fidelity PCR kit and digested with NheI and SwaI (New England Biolabs).

### Transient transfections

All transfections were performed using Lipofectamine 2000 (Thermo Fisher Scientific) with 50 nM Precursor-miR for MIR21 or negative control precursor (Thermo Fisher Scientific). For silencing experiments, SiRNA pools ON-TARGETplus for JAM-A and relative negative controls, miRCURY LNA™ anti-MIR21 or negative control miRCURY knockdown probes (Exiqon, Vedbaek, Denmark) were used at 50 nM. Forty-eight hours post transfection cells were harvested for analyses.

### Nanostring nCounter

nCounter Nanostring (NanoString, Seattle, WA, USA) was used for expression analysis of 770 cancer-related genes with human PanCancer panel (cat GXA-PATH1-12) following the manufacturer’s protocol and conditions. Raw data were log-transformed and normalised by the quantile method after application of a correction factor. Data were filtered to exclude features below the detection threshold in at least half of the samples. *p* values were used to rank RNAs of interest and correction for multiple comparisons was done by the Benjamini–Hochberg method [13].

### Migration assay

Migration assays were performed as previously described [14] in 0.1% of gelatine (Sigma-Aldrich) coated plates. Migration of cells from spheroid to gelatine was monitored at days time points with Celigo®. Analysis was performed on ten replicates for each experiment and in three independent experiments. Area was calculated and difference in migration was calculated as percentage with following formula: (*A*1 − *A*0)/*A*1 × 100 where *A*1 is the calculated area at time point 1 and *A*0 is the area calculated at time 0 (time point of spheroid transfer) for each sample.

### Protein arrays

Global protein expression following JAM-A silencing was performed by means of 660 Human Array (RayBiotech). Six hundred micrograms of total protein extract as previously described [15] was outsourced to RayBiotech. Protein arrays and quantification analysis was performed by RayBiotech as standard company procedure.

Proteins’ phosphorylation status was assessed by mean of R&D Proteome Profiler kit (Bio-Techne, R&D Systems) and Luminex assay (Milliplex, Millipore) as per the manufacturer’s instructions using 600 and 20 µg of total protein, respectively. Data acquisition and quantification analysis were performed by LicoR software for R&D protein array and by Luminex 100 software and Excel spreadsheet for Luminex assay.

### Luciferase assay

JAM-A partial 3′-untranslated region (3′-UTR) was PCR amplified—transcript position 2944-3179 on NM_016946.4—encompassing MIR21 predicted binding site (https://bibiserv2.cebitec.uni-bielefeld.de/rnahybrid [16], and cloned downstream of firefly luciferase gene in the pGL3 modified vector (Promega, Madison, WI, USA) between restriction sites SpeI and SacII. A deletion by site direct mutagenesis kit (Agilent, Santa Clara, USA) was generated (pGL3-JAM-A-3′UTR-Δ21) as per the manufacturer’s instructions. Cells were transfected with 1 µg of pGL3-JAM-A-3′UTR wild-type or pGL3-JAM-A-3′UTR-Δ21 firefly luciferase reporter vector, 0.1 µg of phRL-SV40 renilla control vector (Promega). Firefly and renilla luciferases activities were measured 48 h post transfection by Dual-Luciferase^®^ Reporter DLR™ assay (Promega). Each firefly luciferase emission was normalised to that of renilla for each sample well. Every reaction was performed in triplicate and in three independent experiments.

### In-fusion cloning

Fusion transcripts were generated with In-Fusion^®^ HD Cloning Kit (Takara Bio, Kusatsu, Japan) between JAM-A coding sequence (CCDS1213.1) and its partial 3′UTR or with the 3′UTR with the MIR21 deleted binding site obtained by direct mutagenesis with QuikChange II (Agilent). Transcripts were cloned into pBABE vector with BamHI and SalI (New England Biolabs).

### Retroviral transduction

Retroviral infection of the RKO cell line was achieved by using pBABE-JAM-A CDS WT 3′-UTR. Phoenix-ampho cells were used as packaging cells for retroviral production. After viral infection puromycin (6 µg/ml) was added to the medium for clonal selection for 1 week.

### Immunohistochemistry (IHC)

IHC on TMAs and mice tissues were performed as follows: slides were deparaffinised and rehydrated. Heat-mediated antigen retrieval was performed in sodium citrate buffer (10 mM; 0.05% Tween-20; pH = 6.0) for 25 min at 110 °C. A 3% peroxidase solution was added followed by DAKO blocking reagent for 1 h. Primary antibodies (Online Appendix: Reagents) were applied in DAKO antibody dilution buffer for 1 h at room temperature. Detection was performed with DAKO Envision+ kit (DAKO, Cambridgeshire, UK). Positive and negative controls slides were included in every staining procedure.

### Western blot

Immunoblotting was performed as previously described [12]. Membranes were incubated with primary antibodies (Online Appendix: Reagents), overnight at 4 °C. Then, with relative secondary HRP-conjugated polyclonal goat anti-rabbit or goat anti-mouse antibody (Cell Signalling, Danvers, USA) for 1 h. ECL prime kit (Amersham Biosciences, Chalfont, UK) was used to develop the signal. Standard film and Odyssey Li-COR machine were employed to acquire band images.

### Rap2c activation

RAP2c activation status was assessed with RAP2c kit (Cell Biolabs, CA, USA) as per the manufacturer’s instructions

### Immunofluorescence

Immunofluorescence was performed on cells grown in coverslips and snap frozen embedded PDOs. Cells were fixed with 4% formaldehyde then incubated with blocking buffer solution (5% BSA in PBS) for 1 h. Primary antibodies (Online Appendix: Reagents) were applied in antibody dilution buffer (1% BSA in PBS) for 1 h at room temperature. Secondary antibody Alexa Fluor 488 (Thermo Fisher Scientific) was applied for 1 h. Hoechst 33342 (Sigma-Aldrich, St. Louis, MO) was used for nuclei counterstaining. Vectashield antifade mounting medium (VWR, Leicestershire, UK) was added for reduction of photobleaching. Pictures were acquired with confocal microscope Zeiss-LSM700 at 40× magnification and Zeiss 2009 software (Carl Zeiss, Jena, Germany).

### In situ hybridisation

ISH was performed as we previously described [12, 15].

### DNA extraction, bisulfite sequencing and MS PCR

DNA from cell lines was extracted with Qiagen DNA mini kit (Qiagen, Hilden, Germany) as per the manufacturer’s instructions. DNA was subjected to sodium bisulfite treatment with EZ DNA Methylation Gold kit (ZYMO Research, Irvine, CA, USA). Positive universal methylated DNA and negative un-methylated DNA controls (EpiTect PCR control DNA set; Qiagen) were included in each assay in order to monitor sodium bisulfite conversion efficiency. Converted DNA (20 ng) was used for methylation-specific PCR and bisulfite sequencing.

### Bisulfite sequencing

The region upstream (chr1: 161020929-161021435) of *JAM-A* genomic position containing a CpG island with predicted 41 CpGs was amplified by PCR. Four microliters of PCR template were subjected to TOPO^®^ TA cloning as per the manufacturer’s instructions (Thermo Fisher Scientific). Five representative clones were sequenced by direct Sanger Sequencing (MRC PPU DNA Sequencing and Service, Dundee, UK).

### 5-Aza-2′-deoxycytidine treatment

5-Aza-2′-deoxycytidine (Sigma-Aldrich) was added to cells’ growth medium at a final concentration of 10 μM. As a control, cells treated with DMSO were included. Medium was replaced every day with addition of 5-Aza-2′-deoxycytidine. After 3 days of treatment, cells were recovered for subsequent DNA and RNA analyses.

### Total RNA extraction

Total RNA from cell lines was isolated using TRIZOL method (Invitrogen, Carlsbad, CA, USA). RNA quantity and quality were assessed with Nanodrop2000 spectrophotometer (Thermo Fisher Scientific). Only RNA with an absorbance read ratio 260/280 between 1.8 and 2.0 was used for experiments.

### RT-qPCR

Total RNA was reverse transcribed with microRNA Reverse Transcription Kit (Thermo Fisher Scientific) and expression analysed with TaqMan^®^ assay and probes for hsa-miR-21-5p (Thermo Fisher Scientific) and RNU48 as per the manufacturer’s instructions. For gene expression, High-Capacity RNA-to-cDNA™ Kit (Thermo Fisher Scientific) and SYBR™ Green qPCR with SYBR™ Select Master Mix (Thermo Fisher Scientific) were used. Specific primers were designed for each gene transcript by Primer3 (http://bioinfo.ut.ee/primer3-0.4.0/) and are listed in Online Appendix: Reagents. All reactions, including no-template controls, were run in Bio-Rad C-1000 touch thermocycler (Bio-Rad, Hercules, CA, USA) and Applied Biosystems^®^ StepOnePlus™ Real-Time PCR system (Thermo Fisher Scientific). Each sample was tested in triplicate. Analysis of data was performed with SDS 2.4.1 software (Thermo Fisher Scientific). Relative changes in expression were evaluated by using the 2^ΔCt^ formula.

### Scratch wound assay

Cells were seeded at 3000 cells/well and allowed to reach complete confluence. Following scratch, medium containing doxycycline was replaced and images captured at different days time point with Evos-FL microscope (Thermo Fisher Scientific) or Incucyte S3 (Essen Bioscience, Hertfordshire, UK) until wound closure. Migration capacity was calculated as percentage of wound closure with the following formula: (*D*0 − *D*1)/*D*0 × 100 where *D*1 is the calculated margin distance at time point 1 and *D*0 is the margin distance calculated at time 0 for each sample.

### Growth assays

Growth assays were performed as previously described [14] with 1000 cells/well and Celigo^®^ (Nexcelom Bioscience, Lawrence, MA, USA) cytometer. Scans were repeated at time points and medium with doxycycline was replaced every 48 h. Analysis was performed on 12 replicates for each experiment and in three independent experiments. Spheroids growth was calculated by the average differences in spheroids volumes at time points obtained in μm^3^: *V* = 4/3 π r^3^. Pictures for each well were exported in ImageJ software [17] for analysis.

### Soft agar

Cells (20,000 cells/well) in three replicates were seeded with 20% FBS following mixing and an equal volume of 0.8% agarose (Sigma-Aldrich) solution. The mixture was then poured onto a bed of 1.4% agarose in PBS (FMC BioProducts, Philadelphia, PA, USA). At different time point, pictures were captured with EVOS-FL microscope with phase contrast and RFP channels (Thermo Fisher Scientific). Colonies were then stained and fixed with 0.005% crystal violet (Sigma-Aldrich) and 4% formaldehyde for 1 h.

### Tumour xenograft models

All in vivo experiments were performed in accordance with UK Home Office regulations under the Animals Scientific Procedures Act 1986 and in accordance with UK National Cancer Research Institute guidelines and the ARRIVE guidelines [18]. CRC xenograft tumours were established subcutaneously in 6-7–week-old female nu/nu mice (Charles River, Wilmington, USA) with 2 × 10^6^ cells of Caco-2 TRIPZ CTRL and JAM-A sh in serum-free medium (Gibco) injected in a single flank. CRC xenograft metastatic sites were established intravenously through tail vein in 6–7-week-old female nu/nu mice (Charles River) with 1 × 10^6^ DLD-1 TRIPZ CTRL and JAM-A sh in serum-free medium (Gibco). CRC PDO xenograft tumours were established subcutaneously in 6–7-week-old female NOD-*scid IL2Rg*^*null*^ mice (Charles River) with 1 × 10^6^ cells PDO-TRIPZ-MIR21 and PDO-TRIPZ CTRL cells injected embedded in 100% Matrigel (BD Biosciences) in a single flank. Animals were housed in specific pathogen-free rooms in autoclaved, aseptic microisolator cages with a maximum of five animals per cage. At 9 and 12 weeks post inoculation, mice were culled and primary tumours and lungs removed for paraffin embedding.

### Statistical methods

Survival was calculated from the date of diagnosis to date of death from any cause for overall survival, to the date of CRC-specific death for disease-specific survival and to the date of relapse for relapse-free survival. Differences in survival between patients with low expression and high JAM-A expression were calculated using the Kaplan–Meier method and compared using the log-rank test. For the GSE40966, GSE14333 and TCGA cohorts a survival cut-off was established based on statistical testing. An algorithm separated the samples of a dataset into two groups based on the gene expression.

## Results

### Deregulated JAM-A expression and localisation is a common feature of human CRC

**Fig. 1:**
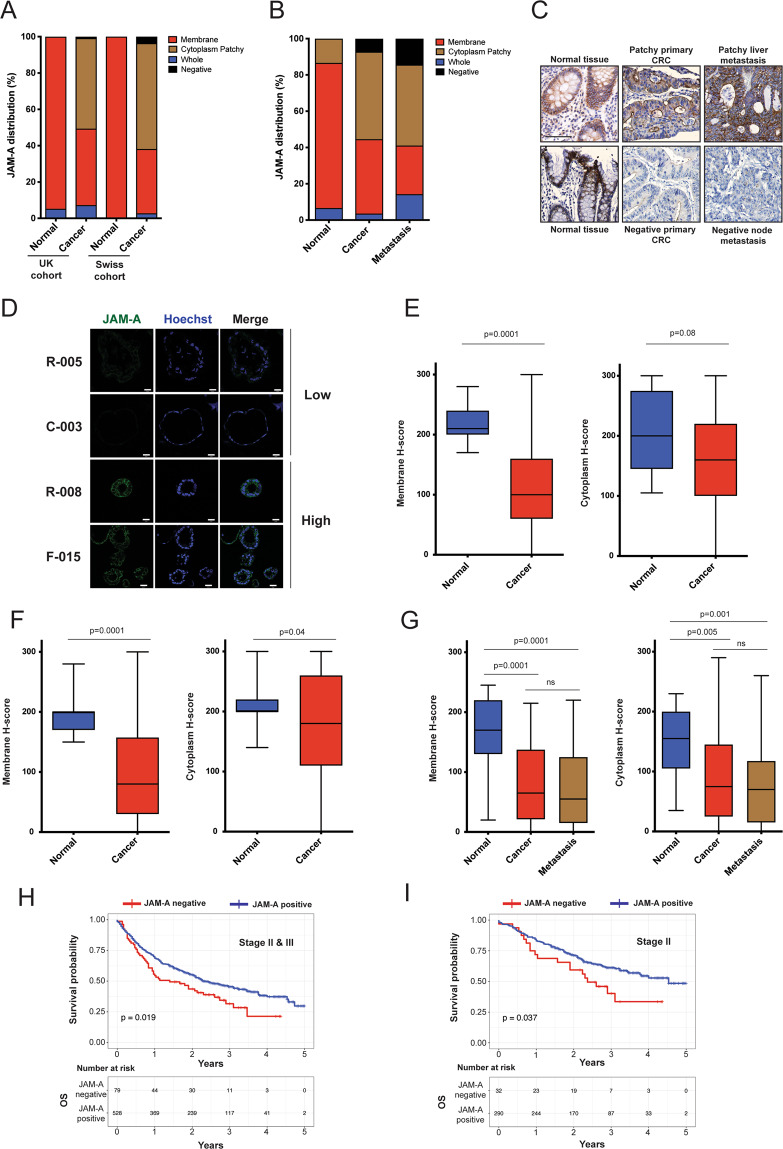
JAM-A expression in colorectal cancer. **A** JAM-A expression and sub-cellular localisation in cancer and normal tissues in the UK (*n* = 266; normal = 19, cancer = 247) and Swiss (*n* = 659; normal = 15, cancer = 644) cohorts; data are shown as percentage. **B** JAM-A expression and sub-cellular localisation in paired normal colon tissue, primary cancer and metastasis, in the Biomax cohort (*n* = 127; normal = 15, cancer = 56; metastasis = 56). **C** Representative images showing patterns of JAM-A expression in normal colon tissue, primary adenocarcinoma and liver metastasis; two representative cases showing JAM-A expression in matched normal tissues (left). Both lesions showed a strong membranous and a moderate cytoplasmic staining. Upper case showed a faint/moderate membranous and a weak cytoplasmic staining (centre top), whereas the liver metastasis was characterised by a faint membranous and a moderate/strong cytoplasmic staining (right top). Lower case showed JAM-A expression loss in both primary and metastatic samples (centre and right bottom). Scale bars 100 μm. **D** JAM-A expression by immunofluorescence staining in PDOs derived from cancer patients. Representative images of low and high expression cases, scale bars 20 μm. Box and whisker plots representing *H*-scores for JAM-A membranous and cytoplasmic staining in normal colon vs cancer in the UK (**E**), Swiss (**F**) [Mann–Whitney test] cohorts and Biomax (**G**) [Kruskal–Wallis test] cohort. Overall survival Kaplan–Meier curves in stages II–III (**H**) and stage II only (**I**) CRC patients in the Swiss cohort, according to JAM-A expression.

JAM-A protein expression was tested by IHC in three independent cohorts of primary and metastatic CRC tissue (Supplementary Fig. S1). In the first series [UK cohort (*n* = 247)], JAM-A staining showed patchy cytoplasmic localisation or complete loss in more than 50% of primary CRC cases. Membranous staining and no cytoplasmic signal were observed in 95% of paired normal tissues and only in 42.1% of CRC cases (Fig. 1A). Similar findings were observed in a second independent set [Swiss cohort (*n* = 644)], with 64% of primary CRC cases showing negative or patchy JAM-A staining, while 100% of matched normal controls exhibiting exclusively membranous staining (Fig. 1A). In the third series [Biomax cohort (*n* = 56)], we tested JAM-A deregulation in the progression from normal to metastatic CRC. JAM-A staining was negative in 7.1% of primary tumours and in 14.3% of paired metastases, while a patchy cytoplasmic expression pattern was observed in 48.2% of primary tumours and in 44.6% of paired metastases (Fig. 1B). Overall, JAM-A expression appeared reduced, scattered across the cytoplasm or completely lost in more than 50% of all cases, both in primary CRC and metastatic deposits (Fig. 1B, C). Supporting these observations, immunofluorescence analysis of ten PDOs from liver, nodal and pelvic metastases of heavily pre-treated gastrointestinal cancer patients [11] showed JAM-A loss in 70% of cases (Fig. 1D and Supplementary Fig. S2A). *H*-score for JAM-A membranous staining was consistently reduced in all three cohorts when primary cancer and metastases were compared to normal tissue (*p* < 0.001) (Fig. 1E–G). Furthermore, a stage dependent effect was observed in localised CRC cases where, membranous JAM-A staining appeared significantly reduced in stage III compared to stage II tumours (Supplementary Fig. S2B). JAM-A loss of expression (defined as *H*-score < 10) correlated with reduced overall survival in stages II and III CRC [HR: 1.43, 95% CI 1.02–2.02, *p* = 0.019 (Fig. 1H)] as well as in stage II only CRC [HR: 1.66, 95% CI 0.90–3.04, *p* = 0.037 (Fig. 1I)] in the Swiss cohort. These findings were further confirmed in 492 patients in three independent cohorts of stage II colon cancer [GSE40966 [19] (*n* = 261); GSE14333 [20] (*n* = 63); TCGA [21] (*n* = 168)] and, taken together, suggest that JAM-A downregulation has a negative prognostic role in early CRC (Supplementary Fig. S2C–E).

### Loss of JAM-A increases the proliferative, migratory and metastatic capacity of CRC

**Fig. 2:**
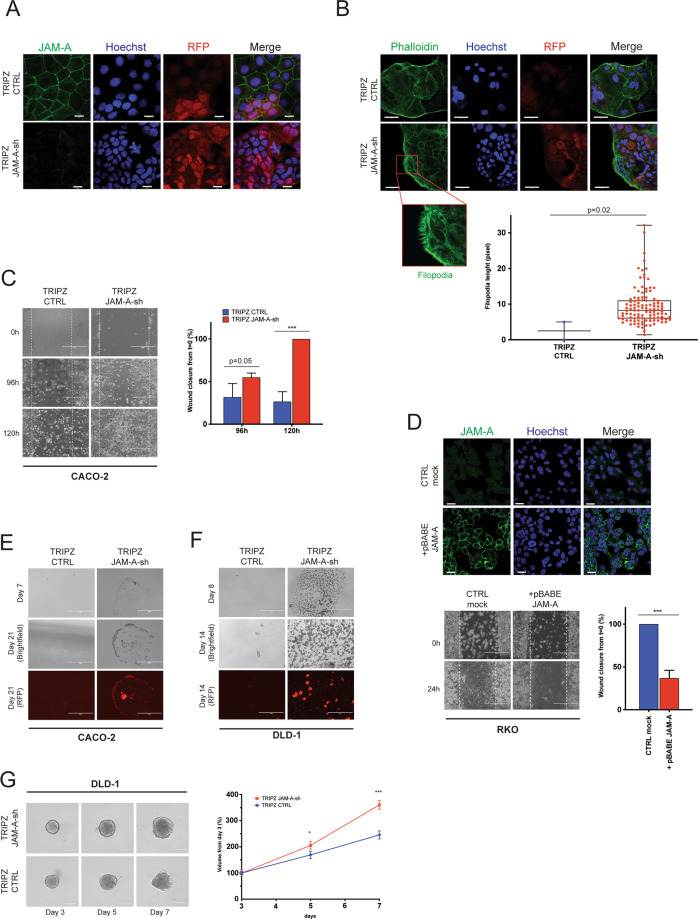
JAM-A downregulation promotes proliferation and migration in CRC. **A** Immunofluorescence staining for JAM-A in Caco-2 cells stably infected with TRIPZ CTRL and TRIPZ JAM-A-sh lentiviral vectors; scale bars 20 μm. **B** Phalloidin staining showing enhanced filopodia formation in Caco-2 cells upon JAM-A downregulation (top) and data quantitation (bottom). Scale bar 50 μm. **C** Scratch wound healing assay using the Caco-2 TRIPZ CTRL and TRIPZ JAM-A-sh clones; quantification data are expressed as average ± SD of three independent experiments (*t*-test, two-sided ***p < 0.001); scale bar 400 μm. **D** Upper panel: immunofluorescence for RKO cells stably infected with a JAM-A-overexpressing (+pBABE-JAM-A) or control (CTRL) retroviral vector; scale bars 20 μm. Lower panel: representative images and quantification of scratch wound healing assay using the CTRL and JAM-A overexpressing RKO clones (*t*-test two-sided, ***p < 0.001); scale bars 400 μm. JAM-A silencing (TRIPZ JAM-A sh) promotes anchorage-independent growth of Caco-2 (**E**) and DLD-1 (**F**) cells in soft agar; scale bars 200 μm. **G** Three-dimensional tumour spheroid formation growth assay upon JAM-A silencing in DLD-1 cells (CTRL *n* = 10, JAM-A sh *n* = 10); scale bars 300 μm. Data are expressed as average ± SD of three independent experiments (*t*-test two-sided, *p < 0.05, ***p < 0.001).

Previous lines of evidence suggest that JAM-A modulation could affect cell motility in breast and gastric cancers [2, 3, 9, 22–24]. In order to investigate whether loss of JAM-A influences cell motility and migration in CRC, we silenced JAM-A in the Caco-2 CRC cell line using a conditional doxycycline-inducible lentiviral JAM-A shRNA silencing construct. Caco-2 cells were chosen as they grow as a 2D monolayer, express relatively high JAM-A mRNA and protein levels (Supplementary Fig. S3A, B) and have been used previously to study JAM-A physiopathology [25]. Conditional JAM-A knockdown caused JAM-A downregulation (Fig. 2A and Supplementary Fig. S3C), promoted a significant increase in filopodia formation (*p*: 0.02) (Fig. 2B) and increased motility by more than 70% after 120 h [(*p* < 0.001) Fig. 2C]. Confirming these data, re-expression of JAM-A by retroviral infection in RKO cells that lack JAM-A due to promoter methylation (Supplementary Fig. S3A, B, D, E) restored JAM-A expression and membranous localisation (Fig. 2D upper panel), and significantly reduced migration compared to control JAM-A-negative RKO cells [37% ± 9% vs 100% ± 0% respectively, (*p* < 0.001) (Fig. 2D lower panel)]. Differences in wound healing ability of the two cell lines were not unexpected and reflected doubling time and migratory potential of the two models [26].

Downregulation of JAM-A also promoted anchorage-independent cell growth in both Caco-2 (Fig. 2E) and DLD-1 (Fig. 2F and Supplementary Fig. S3F, G) cells. The growth promoting effect of JAM-A loss in CRC cells was also confirmed in a tumour spheroid formation assay using the DLD-1 cells that retain the capacity to form tight 3D spheroids in ultra-low attachment plates [14]. Following doxycycline activation, tumour spheroids volume was significantly enhanced in JAM-A-silenced (JAM-A-sh) compared to JAM-A-expressing (CTRL) DLD-1 cells [day 5 JAM-A sh vs CTRL: 205% ± 15% vs 169% ± 14% (*p* < 0.001); day 7 JAM-A sh vs CTRL: 360% ± 16% vs 246% ± 15% (*p* < 0.001); (Fig. 2G)].

**Fig. 3:**
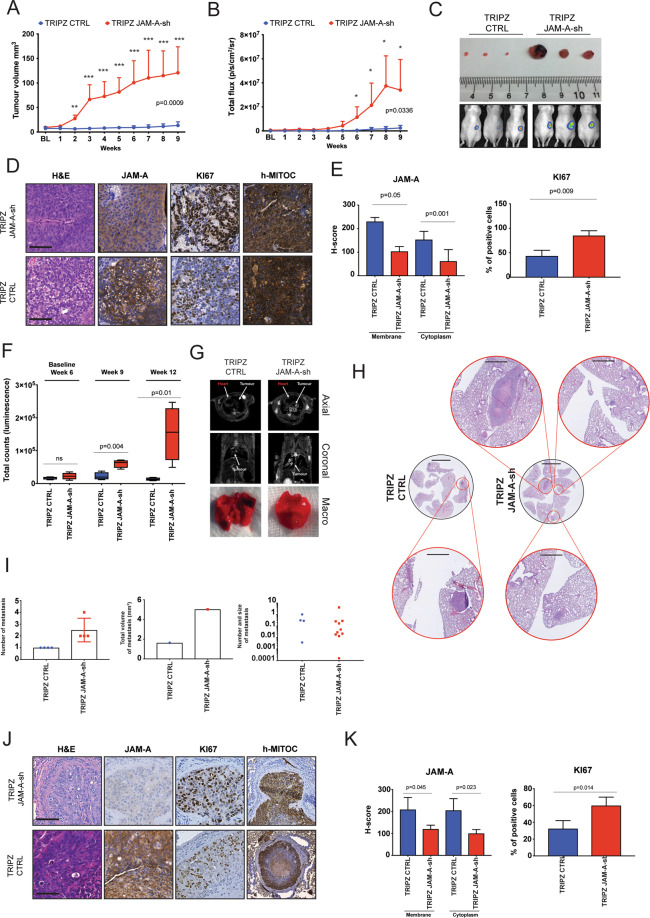
JAM-A downregulation promotes CRC cell growth and metastasis in vivo. Growth of subcutaneous Caco-2 tumours was measured by calliper (**A**) and by in vivo imaging using radiance as endpoint (*t*-test, two-sided *p < 0.05, **p < 0.01, ***p < 0.001) (**B**). **C** Representative examples of explanted tumours and matching in vivo imaging pictures for each of the Caco-2-2 TRIPZ CTRL and TRIPZ JAM-A sh study groups. **D** Representative histology (H&E) and immunohistochemistry for JAM-A and Ki67 along with scoring (**E**) in Caco-2 TRIPZ CTRL and TRIPZ JAM-A sh tumour explants; scale bars 100 μm. **F** Lung colonisation following tail vein injections of the TRIPZ JAM-A sh and TRIPZ CTRL DLD-1 clones was monitored over time using in vivo imaging. **G** Representative magnetic resonance imaging and lung anatomy 12 weeks post tail vein injection of the TRIPZ JAM-A sh and TRIPZ CTRL DLD-1 clones showing effective metastatic deposit formation. **H** Representative histology (H&E) of lung metastatic sites in mice injected with the TRIPZ JAM-A sh and TRIPZ CTRL DLD-1 clones. **I** Absolute number, total volume of metastatic burden and size of individual metastases in mice injected with the TRIPZ JAM-A sh (*n* = 4) and TRIPZ CTRL DLD-1 clones (*n* = 4). **J** Representative histology (H&E) and immunohistochemistry for JAM-A and Ki67 along with scoring (**K**) in DLD-1 TRIPZ CTRL and TRIPZ JAM-A sh lung metastases; scale bars 100 μm.

Next, we assessed the effect of JAM-A downregulation on in vivo tumorigenicity. Luciferase-positive Caco-2 cells expressing JAM-A or control shRNA constructs were implanted in the flank of nude mice (*n* = 4 per group) (Supplementary Fig. S4A) and tumour growth was monitored over a period of 9 weeks using a calliper (Fig. 3A) and luminescence (Fig. 3B). While the tumorigenic potential of Caco-2 shRNA CTRL cells was limited, knockdown of JAM-A significantly increased their ability to form tumours in vivo (Fig. 3A–C). JAM-A knockdown was associated with a significant increase in the proliferation marker Ki67 in Caco-2 tumours (Fig. 3D, E). In order to test whether JAM-A dysregulation also influenced the metastatic potential of CRC cells, we performed tail vein injections of DLD-1 cells expressing the JAM-A or control shRNA constructs and monitored their ability to colonise the lungs over a period of 12 weeks (Fig. 3F and Supplementary Fig. S4B). Tail vein injections were conducted using the DLD-1 line instead of Caco-2, given the latter lack metastatic potential. In vivo imaging, magnetic resonance and macroscopic examination (Fig. 3F–I and Supplementary Fig. S4C) confirmed that JAM-A knockdown promoted the formation of metastatic foci in the lungs. Histology (Fig. 3H) revealed that JAM-A knockdown was associated with an increase in the absolute number of lung metastatic foci, an increase in the total volume of the lung metastatic burden (Fig. 3I) and increased proliferation in metastatic deposits (Fig. 3J, K).

### Loss of JAM-A orchestrates the activation of pro-survival and pro-migratory signalling pathways in CRC

**Fig. 4:**
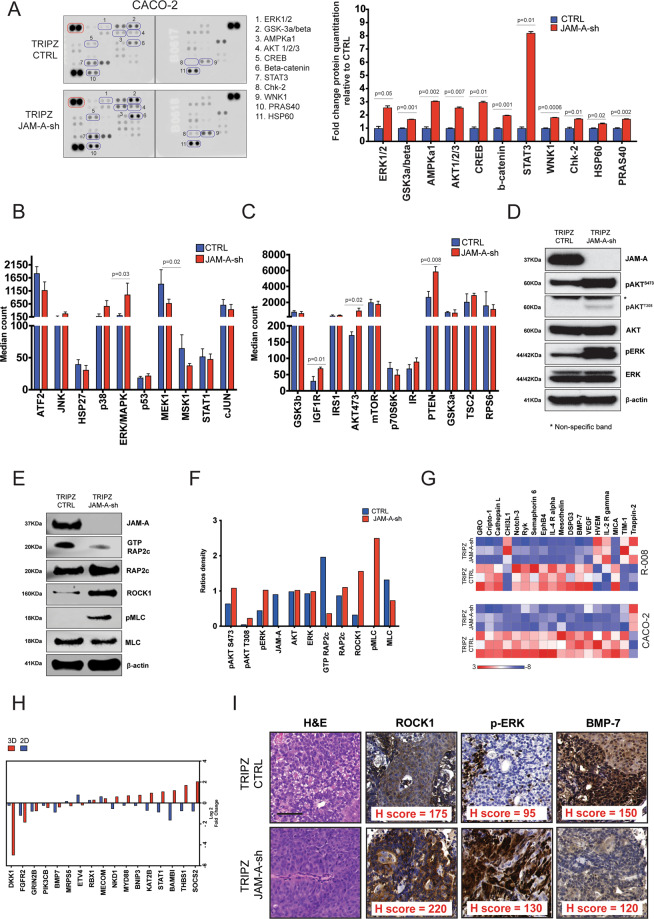
JAM-A silencing activates multiple downstream signalling pathways. **A** Human phospho-kinase array in TRIPZ JAM-A sh and TRIPZ CTRL Caco-2 clones. JAM-A silencing was associated with a significant increase in phosphorylation of the indicated proteins. Blots circled in red on the top left corner represent loading controls proteins. TRIPZ JAM-A sh and TRIPZ CTRL Caco-2 clones were subjected to Luminex assay for phosphoproteins involved in the MAPK/SAPK (**B**) and Akt/mTOR (**C**) pathways. Western blot analysis of the TRIPZ JAM-A sh and TRIPZ CTRL Caco-2 clones showing the effect of JAM-A silencing on the ERK and AKT (**D**) and the ROCK1/pMLC (**E**) signalling pathway. **F** Quantitation of blots in **D** and **E** (three biological replicates). **G** Fold change in common proteins deregulated following JAM-A silencing in 2D (Caco-2) and 3D (R-008 PDO) cellular systems identified with Human Protein 660 plex array (*t*-test, two-sided *p* value < 0.05). **H** Fold change in common genes deregulated following JAM-A silencing in 2D (Caco-2) and 3D (R-008 PDO) cellular systems identified with nCounter NanoString (*t*-test, two-sided *p* value < 0.05). **I** Representative histology (H&E) and immunohistochemistry (with total *H*-scores) for ROCK1, pERK and BMP7 in Caco-2 TRIPZ CTRL and TRIPZ JAM-A sh subcutaneous tumour explants; scale bars 100 μm.

Available literature suggests that JAM-A controls cell migration, proliferation and mitosis through the MAPK/ERK and PI3K/AKT pathways [6, 27, 28]. In order to assess the contribution of JAM-A loss on CRC intracellular signalling, we tested changes in phospho-kinases using a human proteome profiler following conditional JAM-A silencing in Caco-2 (Supplementary Fig. S3C and Fig. 4A) and DLD-1 cells (Supplementary Fig. 3F and Supplementary Fig. S5A). Out of 43 evaluated phosphoproteins, JAM-A silencing was associated with the deregulation of 11 targets in Caco-2 cells (Fig. 4A), and 9 in DLD-1 cells (Supplementary Fig. S5A). In line with previously reported data [6, 27, 28], both ERK1/2 (T202/Y204, T185/Y187) and AKT 1/2/3 (S473) phosphorylation increased by 2.5- and 1.5-fold in Caco-2 and DLD-1, respectively, confirming that JAM-A loss is associated with activation of these two signalling cascades in CRC. A significant increase in ERK and AKT phosphorylation following JAM-A knockdown in Caco-2 cells was further confirmed by cell signalling multiplex luminometric assays (Fig. 4B, C), and by western blotting (Fig. 4D).

Next, we investigated whether PDK1 and mTOR, the two main kinases phosphorylating AKT [29, 30], are involved in the AKT pathway activation in the context of JAM-A downregulation. We starved doxycycline-induced Caco-2 JAM-A-sh cells overnight and subsequently stimulated the cells with insulin, in the absence and presence of either a PDK1 or an mTOR kinase inhibitor (Supplementary Fig. S5B).

Insulin stimulation of the Caco-2 JAM-A-sh cells led to increased AKT phosphorylation in both activating sites. Insulin stimulation failed to induce upregulation of pAKT-Ser476 in the presence of the mTOR kinase inhibitor, suggesting that mTOR is indeed the kinase responsible for the phosphorylation of AKT-Ser476 even in the context of low JAM-A expression. Similarly, insulin stimulation failed to induce upregulation of pAKT-Thr308 in the presence of the PDK1 inhibitor, suggesting that PDK1 mediates phosphorylation of AKT-Thr308 even in the context of low JAM-A expression. Interestingly, whereas the PDK1 inhibitor selectively prevented insulin-mediated upregulation of pAKT-Thr308, the mTOR kinase inhibitor prevented insulin-mediated phosphorylation of both Ser473 and Thr308 sites. A possible explanation for this observation can be that in the Caco-2 JAM-A-sh cells, the phosphorylation of AKT in the Ser473 and Thr308 sites is interdependent, with phosphorylation at the Ser473 site being a pre-requisite for the phosphorylation at the Thr308 site to occur. A similar role for mTOR and PDK1 with regards to AKT activation was also confirmed in the JAM-A-expressing doxycycline-induced Caco-2 CTRL cells (Supplementary Fig. S5B). Overall, our observations suggest that JAM-A modulation does not affect the kinases that directly regulate AKT activation per se; instead, the increased AKT activation observed in the context of low JAM-A may be attributed to other biological processes such as elevated levels of activity of mTORC2 and PDK1 or increased spatial proximity of the aforementioned kinases to AKT.

Given the significant increase in the migratory potential observed following JAM-A silencing in our models and prompted by the physiological role of the JAM-A/RAP2c/ROCK1 signalling axis in epithelial barrier function [31–34], we also tested whether loss of JAM-A may affect motility of CRC cells through the ROCK kinase pathway. Supporting this hypothesis, western blotting analysis showed that upon JAM-A silencing, RAP2c GTP bound form decreased and this resulted in increased levels in ROCK1 and non-muscle myosin 2 (pMLC) phosphorylation (Fig. 4E, F).

In order to further investigate gene and protein expression changes induced by loss of JAM-A, we employed large-scale functional approaches using Caco-2 monolayers grown in 2D, and PDOs established from CRC liver metastases growing in 3D (Fig. 1D and Supplementary 2A). To this end, we interrogated the Caco-2 line and the R-008 PDO (Supplementary Fig. S5C) expressing either the JAM-A or control shRNA constructs using a quantitative multiplexed fluorescent array measuring 660 cytokines. JAM-A downregulation was associated with the deregulation of 207 cytokines (11 upregulated and 196 downregulated) in the Caco-2 cells (Supplementary Table S1) and with the deregulation of 53 cytokines (23 upregulated and 30 downregulated) in the R-008 PDO line (Supplementary Table S2). 18 cytokines were commonly deregulated following JAM-A silencing in both models, with 14 of them showing a concordant up- or downregulation pattern following JAM-A inactivation in both cellular systems (Fig. 4G).

We then analysed the gene-expression profile of the same Caco-2 and R-008 PDO cells expressing either the JAM-A or control shRNA constructs using the NanoString pan-cancer pathway panel: Among the 40 genes significantly deregulated in Caco-2 monolayers, 2 (one upregulated and one downregulated) were dysregulated by more than 2log fold change (Supplementary Tables S3 and 4 and Supplementary Fig. S5D). Among the genes deregulated due to JAM-A inactivation in JAM-A sh 3D organoids, 23 were overexpressed and 24 were downregulated by more than 2log fold change (Supplementary Table S5). Overall, 17 genes were significantly dysregulated both in 2D and 3D with 7 genes showing concordant changes and ten with opposite (Fig. 4H).

Both cytokine and gene-expression analyses showed that bone morphogenetic protein 7 (BMP7), a protein involved in the fine-tuning of the Wnt pathway [35] and a TGFβ1 [36] antagonist, was consistently downregulated following silencing of JAM-A. In agreement with this, analysis of the Caco-2 tumour explants described in Fig. 3A–E confirmed that the silencing of JAM-A also resulted in BMP7 downregulation in vivo (Fig. 4I).

In summary, JAM-A silencing resulted in the activation of pro-survival (AKT, ERK) and pro-migratory (ROCK1) signalling pathways, and may potentiate TGFβ signalling by downregulating the TGFβ1 antagonist BMP7.

### Elevated levels of MIR21 negatively regulate JAM-A mRNA expression in CRC

Experiments in animal models have shown that JAM-A downmodulation follows within 48 h of *Apc* inactivation in GEMMs [10], suggesting that non-genetic mechanisms may underpin the loss of adhesion molecules in CRC progression. Contrary to the epigenetic regulation of JAM-A expression observed in the RKO CRC cells (Supplementary Fig. S3A, B, D, E), analysis of 50 cases of sporadic CRC did not detect *JAM-A* promoter methylation (data not shown), suggesting that the reduction in JAM-A expression observed in CRC is not due to epigenetic silencing of the *JAM-A* promoter.

**Fig. 5:**
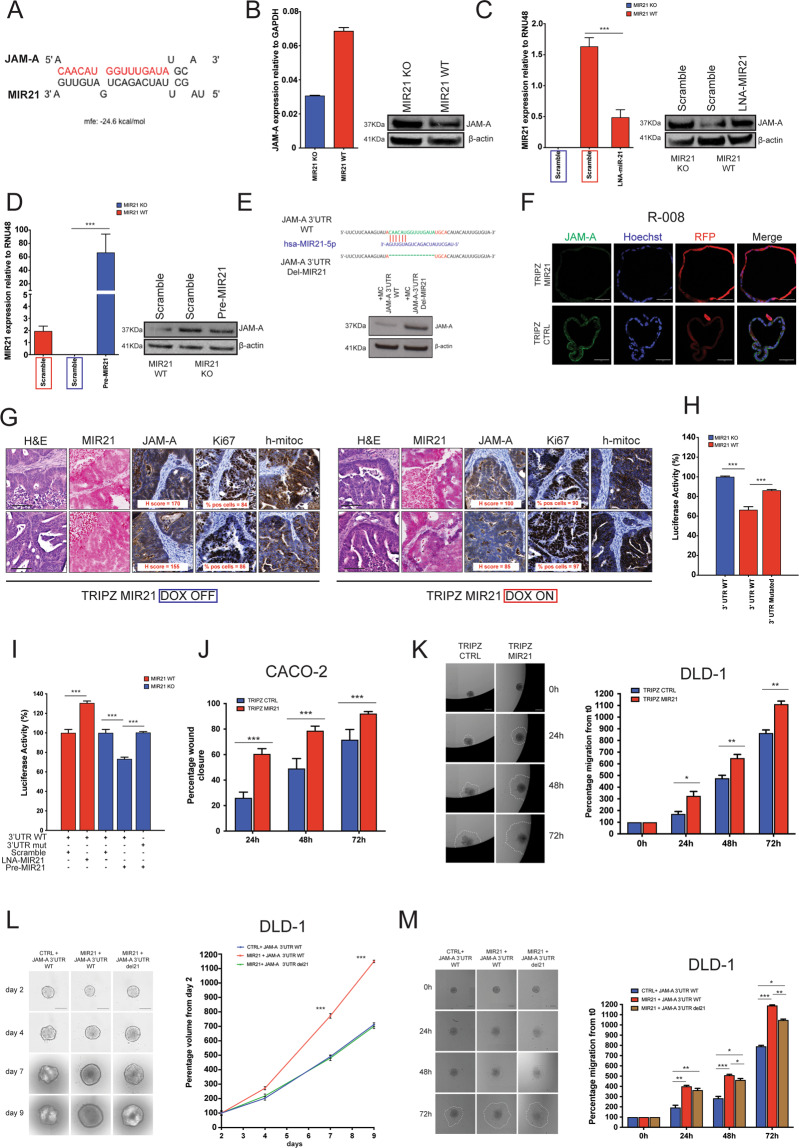
MIR21 regulates expression of *JAM-A* mRNA. **A** In silico prediction of a MIR21 binding site in the *JAM-A* 3′-UTR. Bases highlighted in red indicate the part deleted by site-directed mutagenesis in the luciferase reporter assays described in **H** and **I**. **B** Endogenous JAM-A mRNA and protein expression in the DLD-1 MIR21 isogenic cell system. Effects of MIR21 silencing (**C**) and upregulation (**D**) on JAM-A expression in DLD-1 MIR21^KO^ and MIR21^wt^ cells (*t*-test, two-sided ***p < 0.001). **E** Re-expression of the JAM-A coding sequence in RKO cells, either under the control of the wt JAM-A 3′-UTR (JAM-A 3′-UTR WT) or under the control of an engineered JAM-A 3′-UTR lacking the MIR21 binding site (JAM-A 3′-UTR Del- MIR21). **F** PDOs with high endogenous JAM-A expression were infected with doxycycline-inducible pre-MIR21 (TRIPZ MIR21) or control (TRIPZ CTRL) lentiviral vectors; scale bars 50 μm. **G** Representative histology (H&E), MIR21 expression (in situ hybridisation) and JAM-A and Ki67 expression (IHC) in tumours at end of experiment; scale bars 100 μm. Luciferase reporter assays assessing the impact of endogenous (**H**) or exogenous (**I**) (pre-MIR21; LNA anti-MIR21) MIR21 on the wt or mutant (MIR21 deleted) JAM-A 3′-UTR (*t*-test, two-sided ***p < 0.001). Scratch wound healing assay (**J**) and three-dimensional migration of spheroids through gelatine (**K**) in MIR21-overexpressing cells (TRIPZ MIR21) compared to controls (*t*-test, two-sided *p < 0.05, **p < 0.01, ***p < 0.001). Rescue experiments showing 3D tumour growth (**L**) and 3D migration (**M**) (*t*-test, two-sided *p < 0.05, **p < 0.01, ***p < 0.001) in MIR21-overexpressing DLD-1 spheroids (*n* = 12 each group) transfected with minicircle plasmids containing the JAM-A coding sequence under the control of the intact (wt) *JAM-A* 3′-UTR or under control of *JAM-A* 3′-UTR lacking the MIR21 binding site; scale bars 300 μm.

MicroRNAs (MiRNAs) are frequently upregulated in CRC and contribute to disease progression through the post-transcriptional and translational regulation of various mRNA targets [37]. While exploring whether the reduction in JAM-A expression observed in CRC might be due to MiRNA-mediated silencing, we identified a putative binding site for MIR21, one of the most significantly upregulated MiRNAs in CRC [38], in the *JAM-A* 3′-untraslated region (UTR) (Fig. 5A). To investigate a potential role for MIR21 in the regulation of JAM-A expression, we looked at endogenous JAM-A protein levels in a DLD-1 MIR21 isogenic system [39]; consistent with the notion that MIR21 negatively regulates *JAM-A* expression, we observed higher levels of endogenous JAM-A protein in the DLD-1 MIR21^KO^ cells (Fig. 5B). Interestingly, endogenous *JAM-A* mRNA levels were higher in the DLD-1 MIR21^wt^ cells, suggesting that the MIR21-mediated regulation of *JAM-A* is not due to degradation of the *JAM-A* mRNA but instead due to its translational inhibition (Fig. 5B).

To confirm these observations, we silenced and overexpressed MIR21 in DLD-1 MIR21^wt^ and DLD-1 MIR21^KO^ isogenic cells, respectively. Silencing of MIR21 in DLD-1 MIR21^wt^ cells increased JAM-A protein levels (Fig. 5C), while re-expression of MIR21 in DLD-1 MIR21^KO^ cells decreased JAM-A protein levels (Fig. 5D), indicating that the translation of the *JAM-A* mRNA transcript is regulated by MIR21. A reduction in the JAM-A protein levels following exogenous MIR21 overexpression was also observed in HEK-293T cells (Supplementary Fig. S6A) that express very low levels of endogenous MiRNA [40]. Furthermore, transfection of RKO cells that lack expression of endogenous JAM-A (Supplementary Fig. S3D) with MC plasmids encoding the *JAM-A* coding sequence either under the control of the wt *JAM-A* 3′-UTR or under the control of an engineered *JAM-A* 3′-UTR from which the MIR21 binding site has been deleted (Fig. 5E cartoon), revealed that the *JAM-A* 3′-UTR lacking the MIR21 binding site was more effective in driving JAM-A protein expression (Fig. 5E). Supporting the MIR21-mediated regulation of *JAM-A* mRNA translation observed in CRC cell lines, overexpression of MIR21 in a metastatic CRC PDO line (Supplementary Fig. S6B) also resulted in reduced JAM-A protein levels both ex vivo and in vivo and, despite the small sample size (*n* = 2 per condition), was associated with increased tumour proliferation (Fig. 5F, G and Supplementary Fig. S6C).

The direct binding of MIR21 to the *JAM-A* 3′-UTR was confirmed using luciferase reporter assays in the DLD-1 MIR21 isogenic cell system. Compared to the levels observed in the DLD-1 MIR21^KO^, luciferase activity was suppressed by endogenous MIR21 in the DLD-1 MIR21^wt^ cells when the luciferase gene was under the regulation of the wt *JAM-A* 3′UTR; mutagenesis of MIR21 binding site restored luciferase activity (Fig. 5H). Likewise, exogenous MIR21 up- and downregulation reduced and increased luciferase activity in the DLD-1 MIR21^KO^ and MIR21^wt^ cells, respectively; these effects were abrogated when an engineered version of the *JAM-A* 3′-UTR lacking the MIR21 binding site was used (Fig. 5I). Taken together these observations suggest a direct regulation of *JAM-*A mRNA translation by MIR21 binding to the *JAM-A* 3′-UTR.

Overexpression of MIR21 has been linked to CRC invasion, migration and progression [38]. In order to test whether MIR21 overexpression could recapitulate the phenotype observed upon JAM-A modulation in CRC pre-clinical models (Fig. 2A–G), we generated Caco-2 and DLD-1 cell lines carrying a conditional doxycycline-inducible MIR21 construct and confirmed physiological expression of MIR21 following induction with doxycycline in both systems (Supplementary Fig. S6D, E). In agreement with data observed following JAM-A downregulation in Caco-2 cells (Fig. 2C), MIR21 upregulation also resulted in a significant increase in migration of Caco-2 cells over 3 days (Fig. 5J). Given that 3D assays appear to recapitulate more closely the in vivo cancer physiology [14, 41], we further validated our findings in a 3D migration assay using the DLD-1 cell line and confirmed that MIR21 overexpression induced increased migration also in 3D spheroid conditions (Fig. 5K).

In order to test whether the effect on cell growth and migration observed upon MIR21 overexpression in the DLD-1 cells was due to *JAM-A* downregulation, we performed rescue experiments where the suppression of endogenous *JAM-A* induced by MIR21 overexpression was compensated for by exogenous expression of the *JAM-A* transcript under the control of either a wt 3′-UTR or an engineered 3′-UTR that lacks the MIR21 binding site (Fig. 5E cartoon). MIR21-overexpressing DLD-1 cells co-expressing the *JAM-A* transcript under the control of a wt 3′-UTR proliferated faster compared to MIR21-overexpressing cells co-expressing the *JAM-A* transcript under the control of the mutant 3′-UTR lacking the MIR21 binding site. These observations were consistent with the idea that deletion of the MIR21 binding site in the 3′-UTR of *JAM-A* leads to the abrogation of the MIR21-dependent *JAM-A* downmodulation, resulting in impaired tumour growth and cell migration in 3D. These effects were observed within 5–7 days from MIR21 overexpression for cell proliferation (Fig. 5L) and as early as 24 h for cell migration (Fig. 5M).

Finally, given activating feedforward loops involving miRs and their downstream effectors have been previously described [42], we investigated whether AKT modulation might affect MIR21 and JAM-A expression. However, both insulin and the allosteric pan-AKT inhibitor MK-2206 failed to induce significant or persistent changes in MIR21 expression or JAM-A protein levels in doxycycline-induced JAM-Ash or CTRL Caco-2 cells, making the presence of an activating feedback loop unlikely (Supplementary Fig. S6F, G).

## Discussion

Here, we provide evidence for a functional link between overexpression of the oncogenic MIR21, a hallmark of CRC, reduced expression of the adhesion molecule JAM-A and activation of several oncogenic pathways promoting proliferation, cell migration and disease progression.

We observe that JAM-A downregulation is frequent in CRC and correlates with poor prognosis, particularly in patients with stage II CRC for whom the decision to offer adjuvant treatment is often controversial [43]. From a therapeutic angle, these observations are of particular importance, because they suggest that, contrary to other diseases such as breast cancer and multiple myeloma where JAM-A inhibitors may represent potential therapeutic strategies [7, 44], JAM-A suppression in CRC may have a detrimental effect on patient outcomes. Indeed, JAM-A dysregulation has been observed in multiple tumour types; however, while in breast, lung and head and neck cancers [2–6], JAM-A overexpression enhances proliferation and promotes resistance to targeted therapies [27], in gastrointestinal cancers including gastric and pancreatic neoplasms [8, 9], as well as in thyroid tumours [45], it is JAM-A downregulation that is associated with cancer acceleration and worse prognosis.

In this regard, our study fills in an important gap, because, although the contribution of JAM-A loss in intestinal inflammation is well characterised [46], little is known about its role on CRC tumorigenesis. So far, hints on the involvement of JAM-A in the neoplastic transformation of intestinal cells come from GEMMs, in which conditional inactivation of *Apc* in the large intestine results in rapid JAM-A downregulation, loss of barrier function, penetration of microbiota and activation of an inflammatory response by myeloid cells in the cancer microenvironment [10].

Our data suggest that JAM-A loss has a cell-autonomous, pro-tumorigenic effect of CRC progression that goes beyond the mere permissive effect of eliciting an inflammatory response due to impaired intestinal epithelium barrier function. Indeed, we demonstrate that JAM-A downmodulation retains the ability to promote the CRC phenotype even in absence of microenvironment-related stimuli, an observation in line with data suggesting that JAM-A downregulation in normal fibroblasts accelerates cell proliferation and migration via activation of the MAPK pathway [47].

JAM-A downmodulation follows within hours from Apc loss in CRC animal models [10]. In our three cohorts of human CRC, histological analysis of JAM-A shows reduced or patchy expression in ~40% of cases, with a minority of patients presenting complete JAM-A loss. Altogether, these data suggest that abnormalities in the fine-tuning of JAM-A expression at the post-transcriptional or post-translational level may be responsible for the observed JAM-A downregulation in CRC, rather than genetic or epigenetic events. This hypothesis is supported by the observation that JAM-A promoter methylation was not detected in any of the patients we tested. On the contrary, our data suggest that MIR21 upregulation may be one of the drivers of JAM-A silencing in CRC. We have shown that miR dysregulation plays a critical role in CRC initiation, progression and resistance to anticancer treatments [37, 38, 42]. Here, we demonstrate that JAM-A downregulation is caused, at least in part, by MIR21 overexpression, and that JAM-A rescue significantly abrogates the pro-proliferative and pro-migratory effects of MIR21. Although we provide robust evidence to support a direct regulatory effect of MIR21 on JAM-A expression, we acknowledge that other mechanisms may come into play in controlling adhesion molecules. JAM-A phosphorylation at tyrosine residue Y280 has been reported as a cytokine-induced and SRC-mediated mechanism for barrier function inactivation in human and animal models of colitis [48]. Given MIR21 is involved in the synthesis of numerous inflammatory cytokines such as IL-23 and IL-17A [49] in the colon mucosa, it is possible that a positive feedback loop involving MIR21 overexpression, synthesis of pro-inflammatory factors and activation of oncogenic pathways may also contribute to JAM-A inactivation and the downstream consequences on barrier function and cancer promotion.

The pleiotropic function that JAM-A dysregulation has in CRC compared to other cancer types may relate to tissue-specific differences in transcriptome and miRNAome [50] as well as the intracellular signalling pathways and downstream genes affected by JAM-A downmodulation. For instance, JAM-A deregulation has been shown to activate NFkB [51], MAPK [47, 52] and PI3K [28, 53] signalling cascades in a tissue- and ligand-specific manner, an observation confirmed by our pre-clinical models where loss of JAM-A induced activation of the ERK and AKT pathways.

Although we did not explore any causal relation between β-catenin activation, MIR21 overexpression and JAM-A downregulation, it is noteworthy that, similarly to JAM-A loss [52], MIR21 is also able to promote pSer552 phosphorylation of β-catenin and its nuclear translocation in *APC* mutant colon cancer cells [54]. The β-catenin/TCF activated pathway is known to enhance expression of signal transducer and activator of transcription 3 (STAT3) [55], whose consensus sequence binding sites are present upstream of the pri-MIR21 transcription starting site [56]. Taken together, these observations suggest the presence of a potential feedforward loop, whereby loss of APC and activation of β-catenin may trigger STAT3-mediated overexpression of MIR21, and subsequent MIR21-dependent downregulation of JAM-A; this hypothesis is supported by the fact that two of the CRC cell lines with the highest MIR21 expression (HCT116 and Caco-2) have activating mutations in β-catenin.

A significant downregulation of BMP7 was consistently observed in our models following JAM-A signalling. BMPs attenuate Wnt pathway activity [35, 57] by buffering the nuclear accumulation of β-catenin via PTEN-dependent inhibition of AKT signalling. BMPs are generally downregulated in CRC [58, 59] and BMP signalling is known to counteract TGFβ signalling [57], a previously reported negative regulator of JAM-A [60]. Taken together, these observations suggest that the suppression of BMP7 signalling due to loss of JAM-A may also increase β-catenin activity, thereby further promoting MIR21 transcription, sustaining JAM-A downregulation and promoting CRC progression.

**Fig. 6:**
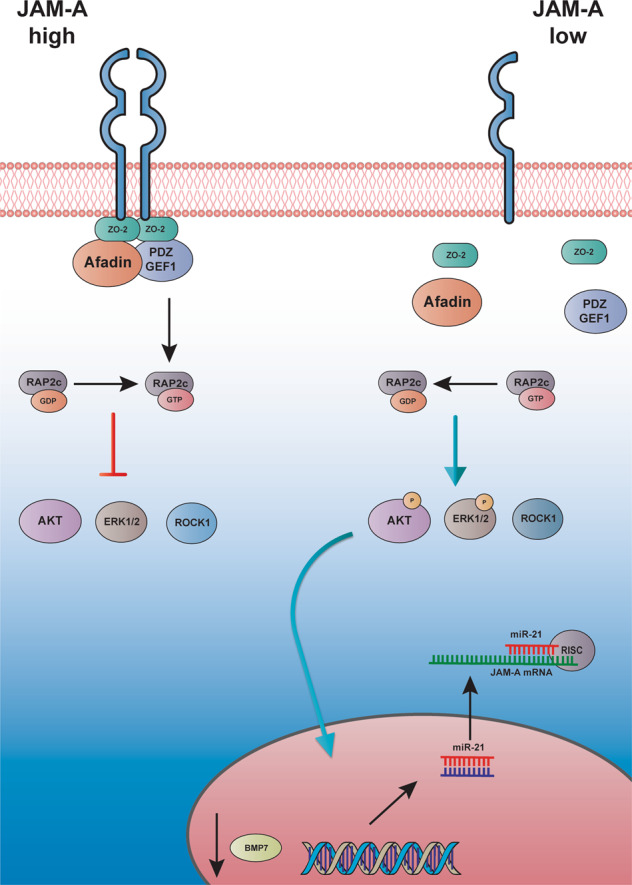
Molecular consequences of JAM-A dysregulation in CRC. MIR21 overexpression causes JAM-A downregulation promoting CRC progression through the activation of MAPK. AKT, ROCK1 pathways and BMP7 inhibition. Suppression of BMP7 signalling may in turn increase β-catenin activity, promote *MIR-21* transcription and sustain the pro-carcinogenetic feedforward loop.

In summary, our data provide novel insights on the cell-intrinsic effect of JAM-A downregulation in colonic tumorigenesis (Fig. 6), and suggest that, beside their well-established role in epithelial barrier function, adhesion molecules also regulate the proliferative and migratory potential of CRC cells by fine-tuning multiple pro-oncogenic signalling cascades.

## Supplementary information

Supplementary Figure S1

Supplementary Figure S2

Supplementary Figure S3

Supplementary Figure S4

Supplementary Figure S5

Supplementary Figure S6

Supplementary Tables

Supplementary Figure Legends

List of Reagents

## Data Availability

NanoString nCounter data have been deposited in the NCBI Gene Expression Omnibus (GEO), with accession code GSE149658.
