# Supplementary figures and images for: MIR21-induced loss of junctional adhesion molecule A promotes activation of oncogenic pathways, progression and metastasis in colorectal cancer
Source: Cell Death Differ. 2021 Jul 5;28(10):2970–82. doi: 10.1038/s41418-021-00820-0 (PMC8481293; doi:10.1038/s41418-021-00820-0)

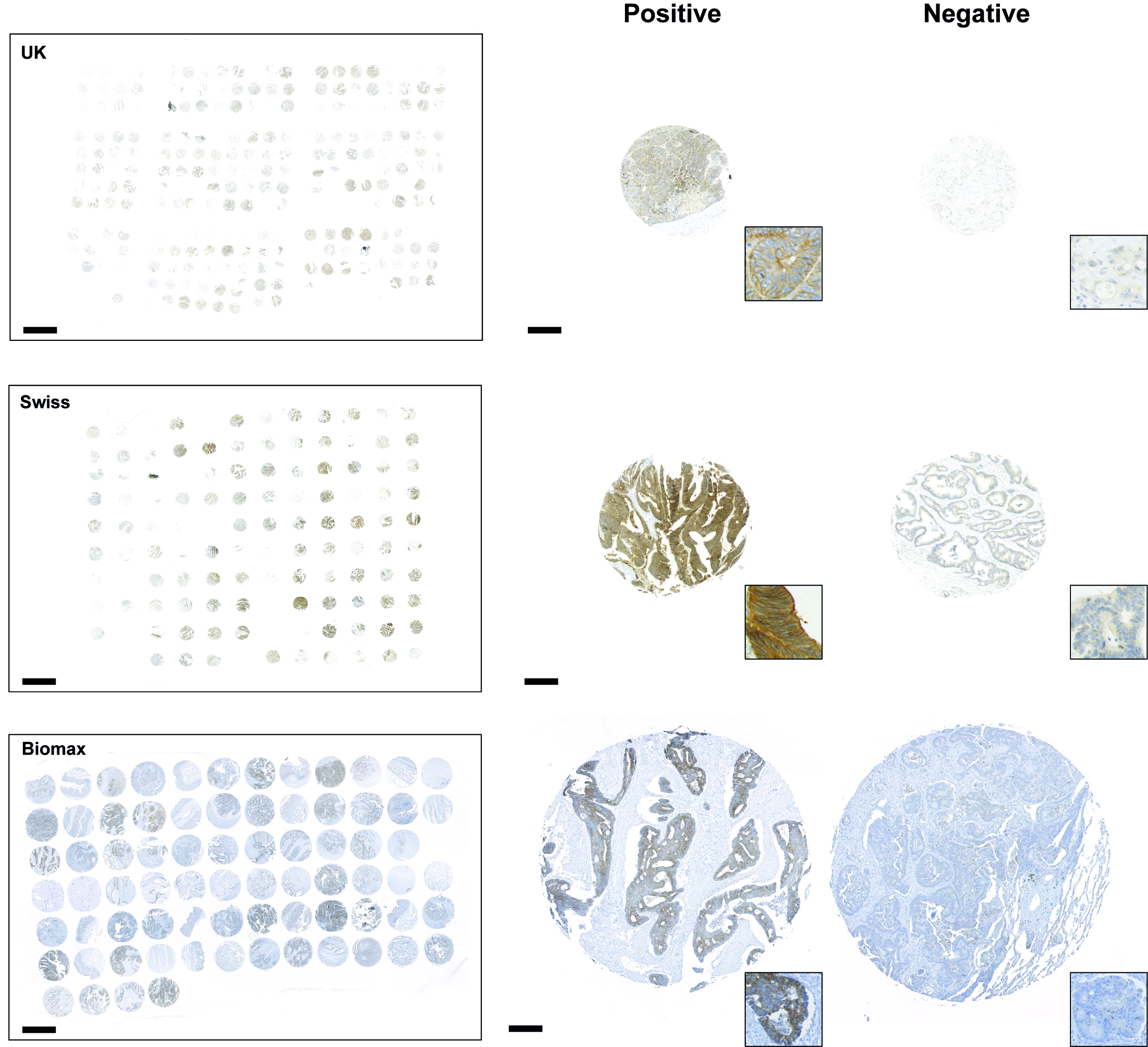

Supplement: Supplementary file 1 — Supplementary Figure S1 [file 41418_2021_820_MOESM1_ESM.tif]

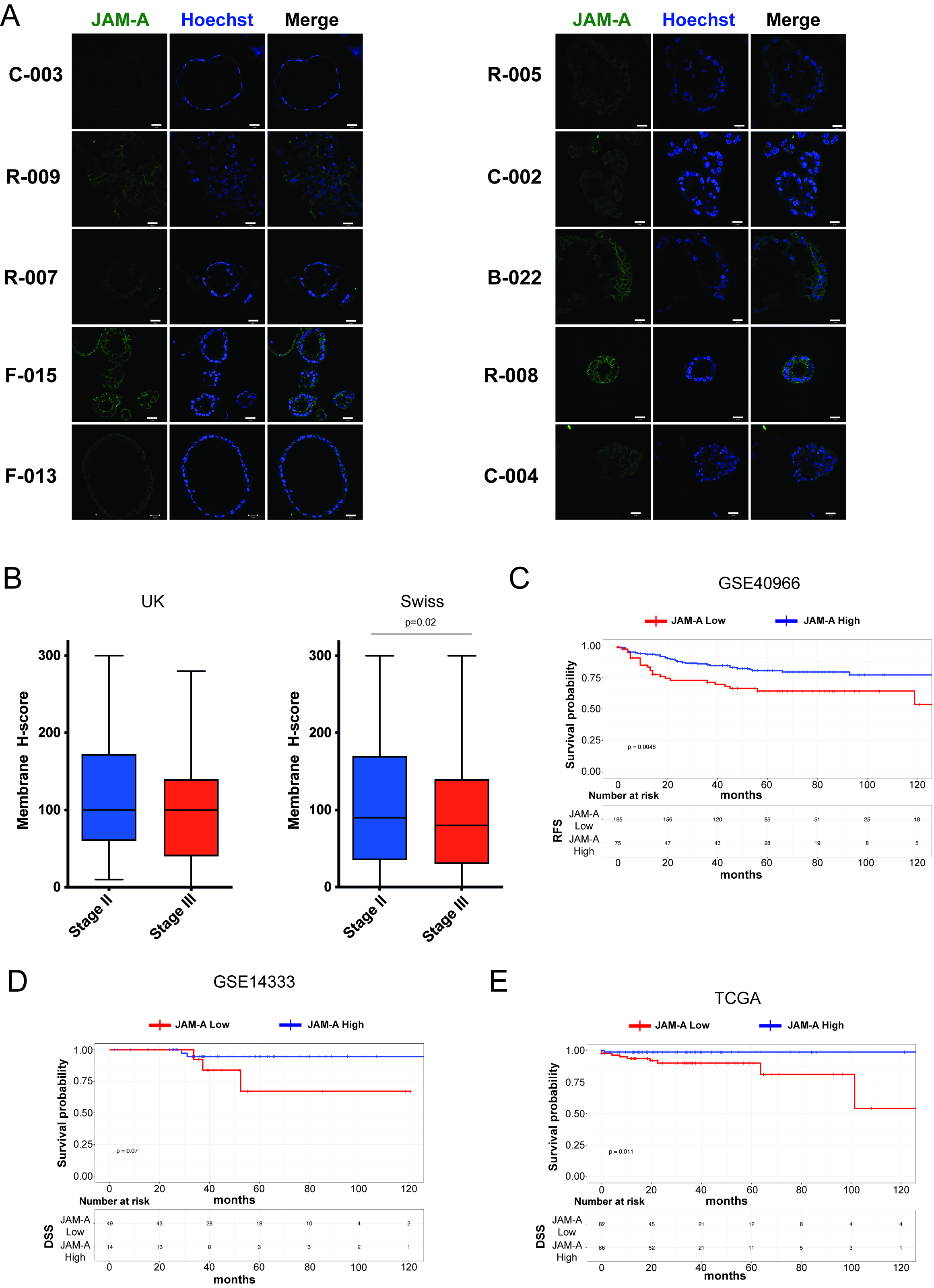

Supplement: Supplementary file 2 — Supplementary Figure S2 [file 41418_2021_820_MOESM2_ESM.tif]

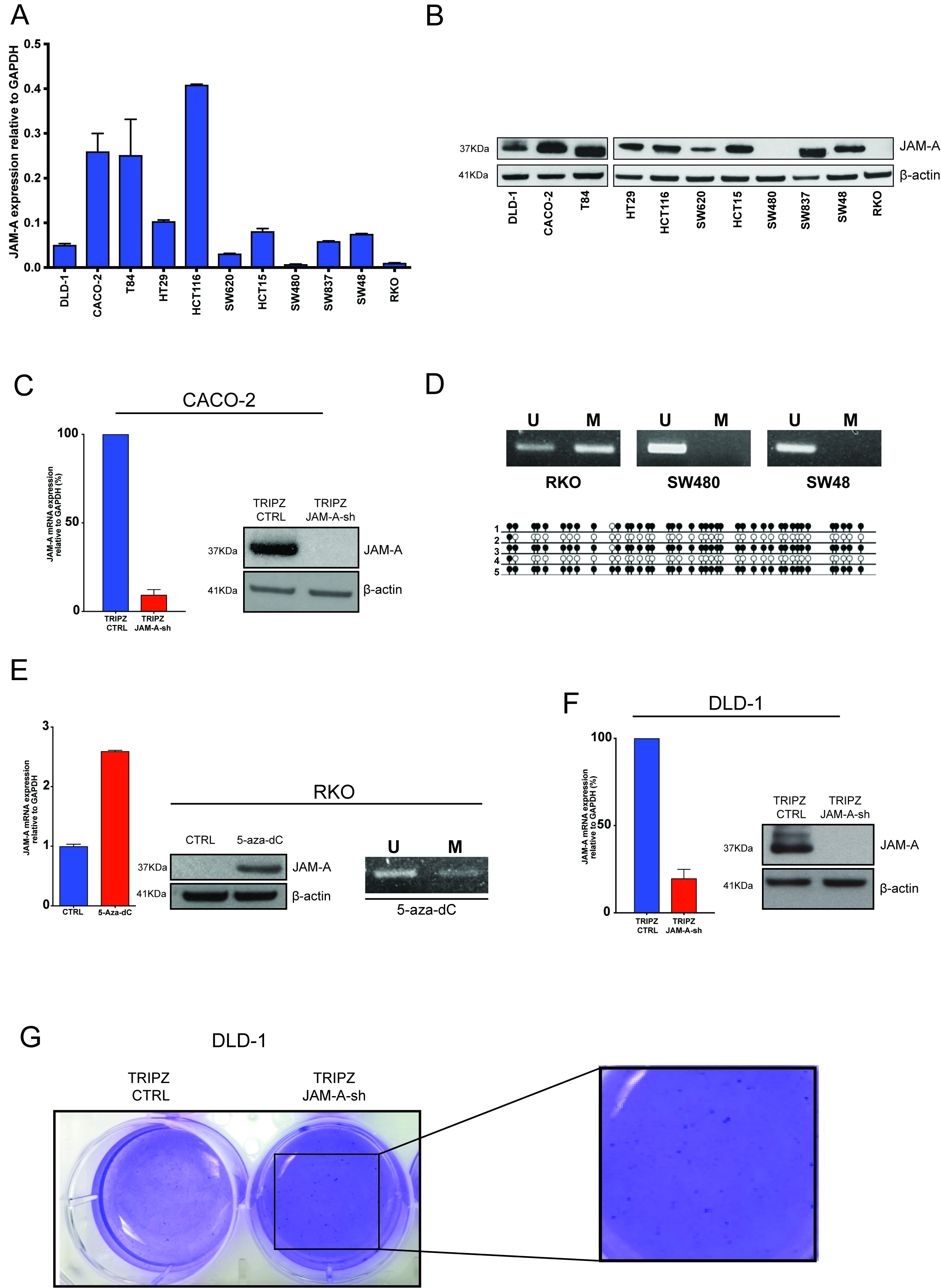

Supplement: Supplementary file 3 — Supplementary Figure S3 [file 41418_2021_820_MOESM3_ESM.tif]

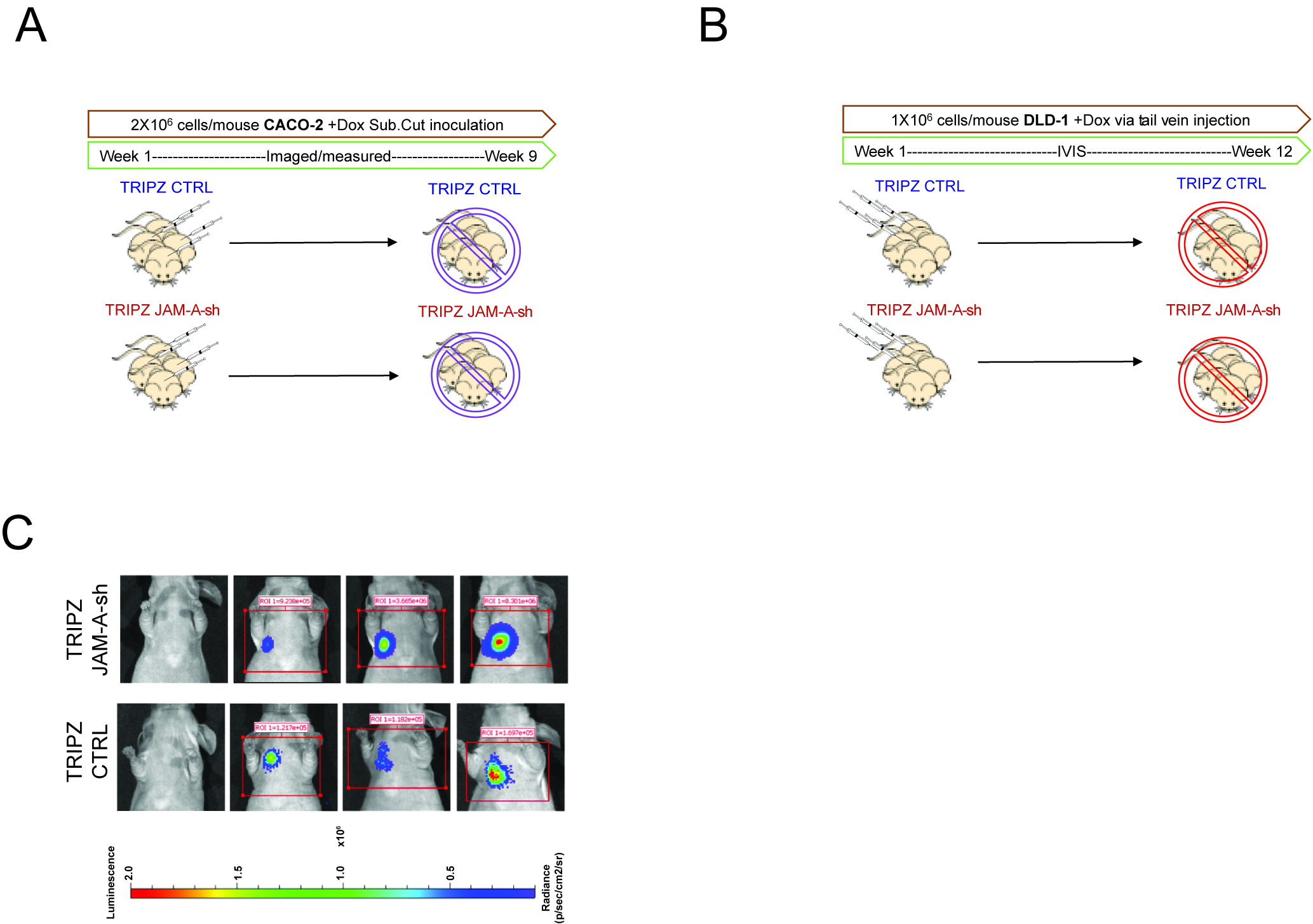

Supplement: Supplementary file 4 — Supplementary Figure S4 [file 41418_2021_820_MOESM4_ESM.tif]

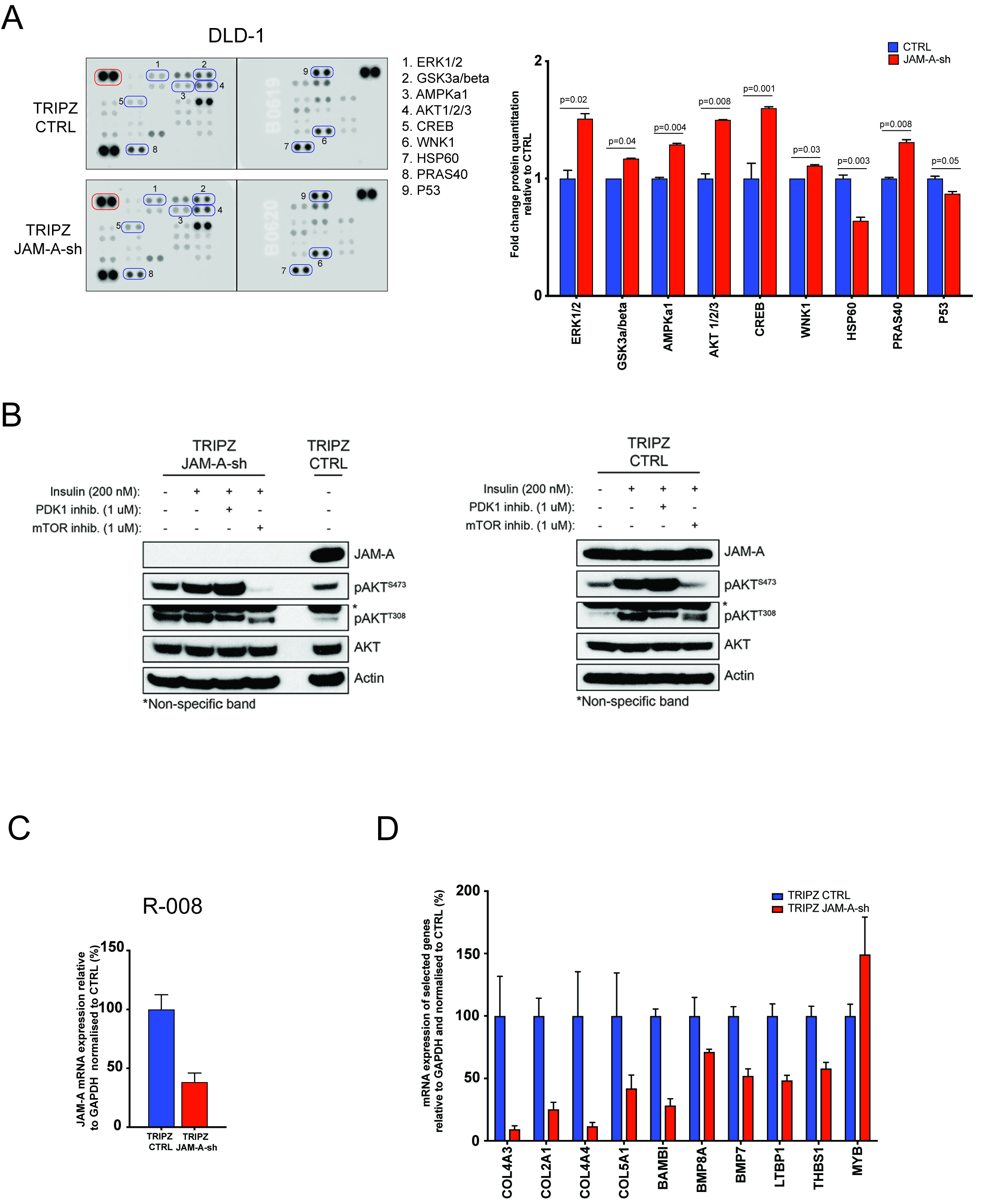

Supplement: Supplementary file 5 — Supplementary Figure S5 [file 41418_2021_820_MOESM5_ESM.tif]

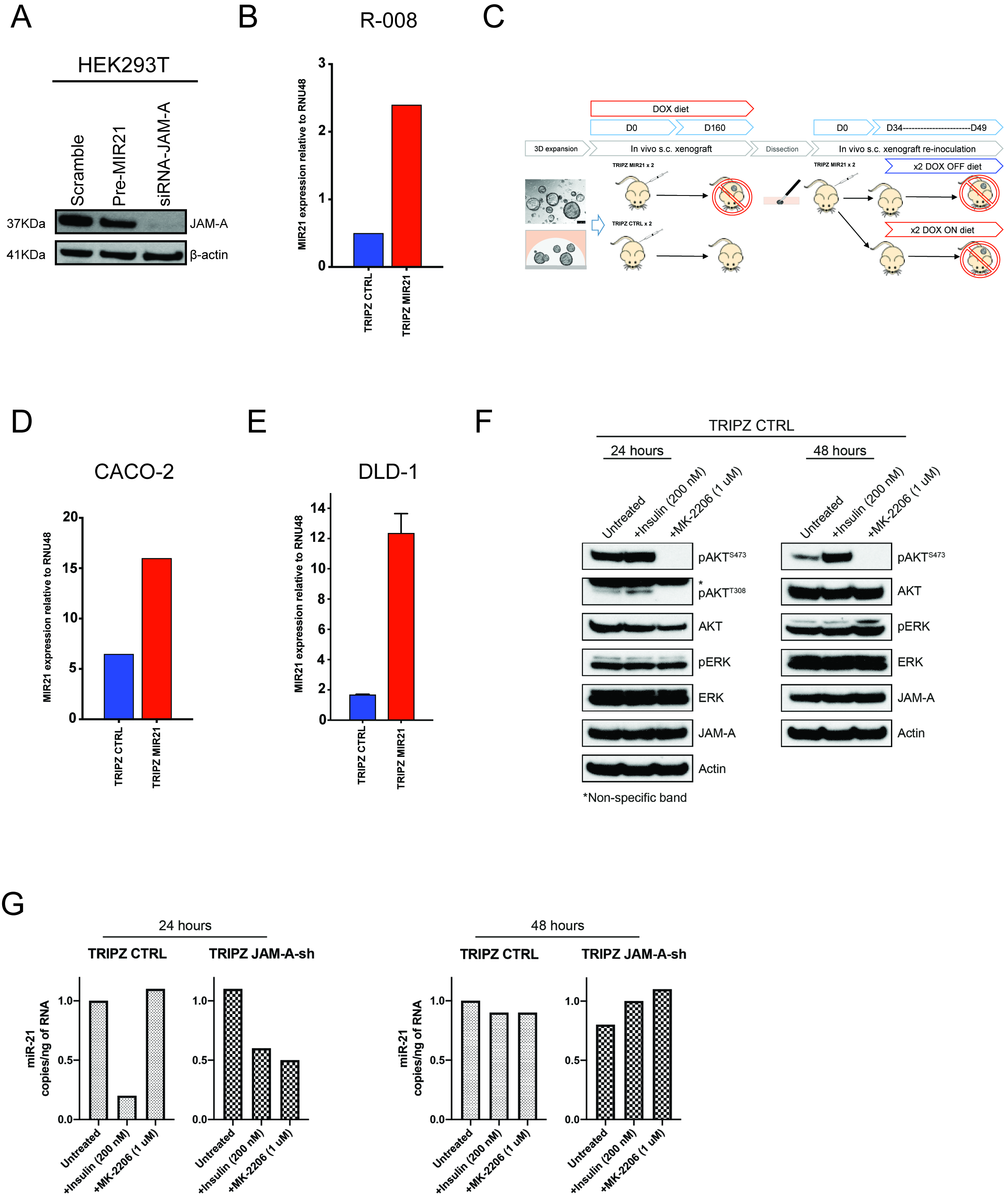

Supplement: Supplementary file 6 — Supplementary Figure S6 [file 41418_2021_820_MOESM6_ESM.tif]
