# Supplementary material for: MIR21-induced loss of junctional adhesion molecule A promotes activation of oncogenic pathways, progression and metastasis in colorectal cancer
Source: Cell Death Differ. 2021 Jul 5;28(10):2970–82. doi: 10.1038/s41418-021-00820-0 (PMC8481293; doi:10.1038/s41418-021-00820-0)
Supplement: Supplementary file 8 — Supplementary Figure Legends [file 41418_2021_820_MOESM8_ESM.docx]

**Supplementary Figure S1. Expression of JAM-A in primary and metastatic CRC.** Representative TMAs obtained from three different sources and showing a comparable pattern of JAM-A staining (scale bars 2.5 mm). For each TMA a JAM-A positive and a JAM-A negative cases are shown (scale bars 250 um). Positive cases: upper panel moderate/strong membranous and moderate cytoplasmic; middle panel moderate membranous and strong cytoplasmic; lower panel moderate membranous and moderate/strong cytoplasmic.

**Supplementary Figure S2. Expression of JAM-A in mCRC PDOs and association with survival outcome in CRC patients.** (***A***) JAM-A expression by immunofluorescence staining in ten (all cases, including reported examples in Fig.1) PDOs derived from gastric and mCRC patient, scale bar 20μm. (***B***) JAM-A H-score by stage in the UK and Swiss cohorts. (***C***) Kaplan-Meier curves for Relapse Free Survival (RFS) in the GSE40966 cohort (n=261). Disease-specific survival (DSS) in the GSE14333 (n=63) (***D***), and TCGA (n=168) (***E***) cohorts.

**Supplementary Figure S3**. **JAM-A expression and modulation in CRC cell lines.** (***A***) qPCR analysis for JAM-A mRNA expression (relative to GAPDH) in a panel of CRC cell lines; data are expressed as average ±SD of three independent experiments. (***B***) JAM-A protein expression in a panel of CRC cell lines. (***C***) JAM-A mRNA and representative protein expression in CACO-2 cells stably infected with TRIPZ CTRL and TRIPZ JAM-A sh lentiviral vectors; data are expressed as average ±SD of three independent experiments. (***D***) Methylation-specific PCR in RKO cells revealed methylation of the *JAM-A* CpG island promoter region. SW48 cell lines were used as negative controls. Lollipop representation (black: methylated CpG; white: unmethylated CpG) of bisulfite sequencing of cloned CpG island in RKO cells: five representative clones were sequenced following cloning of the PCR fragment. (***E***) Treatment of RKO cells were with 10μM of 5-aza-2´-deoxycytidin for 72 hours induced the de-methylation of the *JAM-A* promoter and increased JAM-A mRNA and protein expression levels. (***F***) JAM-A mRNA and protein expression in DLD-1 cells stably infected with TRIPZ CTRL and TRIPZ JAM-A sh lentiviral vectors; data are expressed as average ±SD of three independent experiments. (***G***) Crystal violet staining of DLD-1 TRIPZ CTRL and TRIPZ JAM-A sh clones grown in soft agar shows few colonies growing in DLD-1 TRIPZ CTRL compared to several colonies in TRIPZ JAM-A sh (dark pink).

**Supplementary Figure S4. JAM-A modulation *in vivo*.** (***A***) Schematic representation of the subcutaneous xenograft in vivo study using Luc+ve CACO-2 cells stably infected with the TRIPZ CTRL (n=4) and TRIPZ JAM-A sh (n=4) lentiviral constructs. (***B***) Schematic representation of the lung colonization in vivo study using Luc+ve DLD-1 cells stably infected with the TRIPZ CTRL and TRIPZ JAM-A sh lentiviral constructs. (***C***) Representative images of in vivo imaging, number in red squares indicate total flux (p/s/cm2/sr).

**Supplementary Figure S5.** **Effect of JAM-A inhibition on downstream pathways.** (***A***) Human Phospho-Kinase Array in the TRIPZ JAM-A-sh and TRIPZ CTRL DLD-1 clones (left panel). JAM-A silencing (TRIPZ JAM-A-sh) was associated with a significant increase in phosphorylation of the indicated proteins: ERK1/2 (T202/Y204,T185/Y187), GSK3a/beta(S21/S9), AMPKa1 (T183), AKT 1/2/3 (S473), CREB (S133), WNK1(T60), HSP60 (total), PRAS40 (T246), P53 (S15) (right panel). Blots circled in red on the top left corner represent loading controls proteins. (***B***) DOX-induced CACO2 JAM-A-sh and CTRL cells were starved overnight (0.1% FBS) and subsequently stimulated with Insulin (200 nM) for 20 min, in the absence and presence of either a PDK1 inhibitor (GSK2334470; 1 uM) or an mTOR kinase inhibitor (KU-0063794; 1 uM). (***C***) JAM-A mRNA expression in R-008 PDOs stably infected with TRIPZ CTRL and TRIPZ JAM-A sh lentiviral vectors. Every reaction was performed in triplicate (***D***) Validation (qPCR) of ten genes identified by the nCounter Nanostring analysis (**Supplementary Table S5**) in the TRIPZ JAM-A-sh and TRIPZ CTRL CACO-2 clones; gene expression data are normalised to expression levels in CACO-2 TRIPZ CTRL cells. Every reaction was performed in triplicate.

**Supplementary Figure S6.** **miR-21 controls JAM-A expression.** (***A***) Representative blot showing transient over-expression of MIR21 in HEK293T cell line that reduces JAM-A protein levels; a siRNA against JAM-A mRNA was used as positive control. (***B***) MIR21 expression levels (qPCR) in R-008 PDOs lines stably infected with a doxycycline-inducible MIR21 over-expressing (TRIPZ MIR21) or control (TRIPZ CTRL) lentiviral vector. (***C***) PDOs described in (***B***) were implanted sub-cutaneously in the flank of nude mice (n=2 each group) and allowed to grow while the mice received a doxycycline-containing diet. Once tumours reached a critical mass, (TRIPZ MIR21) mice were randomised and continued either on a doxycycline-containing (DOX ON) or on a doxycycline-free (DOX OFF) diet. MIR21 expression levels (qPCR) in CACO-2 (***C***) and DLD-1 (***D***) cell lines stably infected with a doxycycline-inducible MIR21 over-expressing (TRIPZ MIR21) or control (TRIPZ CTRL) lentiviral vector. Effects of AKT pathway modulation on JAM-A (***F***) and MIR21 expression (***G***). DOX-induced CACO2 CTRL cells were treated with Insulin (200 nM) or with the allosteric pan-AKT inhibitor MK-2206 (1 uM), and protein lysates were collected and tested by western-blots at 24h- and 48h-post AKT stimulation/inhibition (***F***). MIR21 copies by digital-droplet PCR in individual experiments compared to respective untreated CACO-2 CTRL conditions (***G***).
